# *Sauropus brevipes* ethanol extract negatively regulates inflammatory responses *in vivo* and *in vitro* by targeting Src, Syk and IRAK1

**DOI:** 10.1080/13880209.2020.1866024

**Published:** 2021-01-13

**Authors:** Ji Hye Kim, Jae Gwang Park, Yo Han Hong, Kon Kuk Shin, Jin Kyeong Kim, Young-Dong Kim, Ki Dong Yoon, Kyung-Hee Kim, Byong Chul Yoo, Gi-Ho Sung, Jae Youl Cho

**Affiliations:** a Department of Integrative Biotechnology and Biomedical Institute for Convergence at SKKU (BICS), Sungkyunkwan University, Suwon, Republic of Korea; b Division of Translational Science, Research Institute, National Cancer Center, Goyang, Republic of Korea; c Department of Life Science, Hallym University, Chuncheon, Republic of Korea; d College of Pharmacy, The Catholic University of Korea, Bucheon, Republic of Korea; e Proteomic Analysis Team, Research Institute, National Cancer Center, Goyang, Republic of Korea; f Institute for Bio-Medical Convergence, International St. Mary’s Hospital and College of Medicine, Catholic Kwandong University, Incheon, Republic of Korea

**Keywords:** Natural product medicine, anti-inflammatory remedy, flavonoids, gastritis, peritonitis

## Abstract

**Context:**

*Sauropus brevipes* Müll. Arg. (Phyllanthaceae) has been used as an effective ingredient in a decoction for the treatment of diarrhoea. However, there was no report on its modulatory role in inflammation.

**Objective:**

This study investigates anti-inflammatory effect of *S. brevipes* in various inflammation models.

**Materials and methods:**

The aerial part of *S. brevipes* was extracted with 95% ethanol to produce Sb-EE. RAW264.7 cells pre-treated with Sb-EE were stimulated by lipopolysaccharide (LPS), and Griess assay and PCR were performed. High-performance liquid chromatography (HPLC) analysis, luciferase assay, Western blotting and kinase assay were employed. C57BL/6 mice (10 mice/group) were orally administered with Sb-EE (200 mg/kg) once a day for five days, and peritonitis was induced by an intraperitoneal injection of LPS (10 mg/kg). ICR mice (four mice/group) were orally administered with Sb-EE (20 or 200 mg/kg) or ranitidine (positive control) twice a day for two days, and EtOH/HCl was orally injected to induce gastritis.

**Results:**

Sb-EE suppressed nitric oxide (NO) release (IC_50_=34 µg/mL) without cytotoxicity and contained flavonoids (quercetin, luteolin and kaempferol). Sb-EE (200 µg/mL) reduced the mRNA expression of inducible NO synthase (iNOS). Sb-EE blocked the activities of Syk and Src, while inhibiting interleukin-1 receptor associated kinases (IRAK1) by 68%. Similarly, orally administered Sb-EE (200 mg/kg) suppressed NO production by 78% and phosphorylation of Src and Syk in peritonitis mice. Sb-EE also decreased inflammatory lesions in gastritis mice.

**Discussion and conclusions:**

This study demonstrates the inhibitory effect of Sb-EE on the inflammatory response, suggesting that Sb-EE can be developed as a potential anti-inflammatory agent.

## Introduction

Inflammation, a non-specific response that is part of innate immunity, occurs as a protective response that does not depend on the type of pathogen or previous infection (Chen et al. 2018). This response is initiated when pathogens are recognized by pathogen recognition receptors (PRRs). Pathogens such as bacteria, fungi and viruses interact with different PRRs, including Toll-like receptors (TLRs), retinoic acid-inducible gene-I-like receptors (RLRs), nucleotide-binding oligomerization domain-like receptors (NLRs) and C-type lectin receptors (CLRs) (Takeuchi and Akira 2010). Lipopolysaccharides (LPSs), a component of the cell wall in Gram negative bacteria, are recognized by TLR4, which resides on the surface of macrophages and transmits inflammatory signals inside the cells (Sabroe et al. 2008). LPS-stimulated TLR4 recruits myeloid differentiation primary response 88 (MyD88) and TIR-domain-containing adapter-inducing interferon-β (TRIF), known as adaptor molecules, and activates nuclear factor-kappa B (NF-κB) and activator protein 1 (AP-1) by mediating phosphorylation-dependent signalling enzyme cascades (Akira and Takeda 2004; Kagan and Medzhitov 2006; Kagan et al. 2008; Tanimura et al. 2008). In NF-κB activation, phosphorylation and degradation of inhibitor of nuclear factor kappa B (IκBα) are central steps, and this reaction is facilitated by activation of Src, Syk and p85 (Li et al. 2003; Kawai and Akira 2007; Lee et al. 2007). Meanwhile, AP-1 signalling is mainly activated by sequential phosphorylation of signalling kinases such as interleukin-1 receptor associated kinases (IRAKs) and mitogen-activated protein kinases (MAPKs) such as extracellular-signal-regulated kinase (ERK), c-Jun N-terminal kinase (JNK) and p38 (Janssens and Beyaert 2003). Then, the activated transcription factors (NF-κB and AP-1) modulate expression of cytokines and inflammatory genes, including inducible nitric oxide synthase (iNOS), cyclooxygenase-2 (COX-2) and tumour necrosis factor-α (TNF-α), thereby treating infectious lesions or repairing tissue (Baeuerle and Henkel 1994; Liu et al. 2018). However, excessive inflammation can cause tissue damage or chronic inflammatory diseases. Chronic inflammatory diseases such as ulcerative colitis, rheumatoid arthritis, lupus and Crohn’s disease are painful and can adversely affect quality of life; they may also serve as the precursors to cardiovascular disease, diabetes and cancer (Roifman et al. 2011; Schett et al. 2013). Therefore, after the danger signals such as pathogens and irritants are eliminated, post-inflammatory resolution is critical (Schett and Neurath 2018).

Previous drugs have targeted a single protein or enzyme, thereby reducing off-target effects and minimizing side effects. However, given the increasing evidence that chronic and intractable diseases – including chronic inflammatory diseases, neurodegenerative diseases and cancer – are caused by multiple genetic or environmental factors, the development of multi-target medications is accelerating (Medina-Franco et al. 2013; Kumar et al. 2018). In particular, ingredients extracted from natural products are in the spotlight in the multi-drug development field due to their therapeutic efficacy and safety, which has been proven by their longstanding usage as herbal medicines (Hoffmann 2013). For example, stillen was developed using ingredients from *Artemisia asiatica* Nakai (Asteraceae) extract and is currently on sale as an anti-inflammatory agent against gastritis (Park et al. 2008; Yoon et al. 2011). Mevalotin, which is used for treatment of hyperlipidaemia, and tacrolimus, an immunosuppressive agent, are both medicines based on natural products (Calixto 2019). The natural product library is an interesting source for drug development, so continuous efficacy evaluation is required.

The genus *Sauropus* (Phyllanthaceae) consists of 40 species which are mainly distributed in Southeast Asia, Malaysia and Australia. Of these, *S. androgynous* L. Merr., which is an ingredient of Jamu, has traditionally been used for treating genito-urinary diseases, cardiovascular diseases and diabetes, as well as for enhancement of breast milk production and eyesight (Zhang et al. 2020). Jamu is a traditional Indonesian medicine consisting of natural ingredients such as roots, bark, flowers, seeds, leaves and fruits (Woerdenbag and Kayser 2014). Leaves of *S. androgynous* contain phytochemicals such as polyphenols, anthocyanins, carotenoids, ascorbic acids and tannins (Singh et al. 2011). In addition, *S. androgynous* has a high content of flavonoids including quercetin, kaempferol, myricetin, luteolin and apigenin (Arif 2020). It has been reported that ethanol, methanol or aqueous extracts of *S. androgynus* have anti-inflammatory, antimicrobial and antioxidant activities *in vitro* (Eng Khoo et al. 2015), and these pharmacological properties are thought to be due to bioactive phytochemicals. The anti-inflammatory properties of *S. rostratus* Miq. have also been reported (Zhen et al. 2013). *S. brevipes* has been used to treat diarrhoea in a decoction, but no literature reports the pharmacological activity of this plant. Therefore, this study investigates the anti-inflammatory activity and mechanism of action of *S. brevipes* ethanol extract (Sb-EE) in LPS-stimulated RAW264.7 cells, LPS-induced peritonitis mice and HCl/EtOH-triggered gastritis mice.

## Materials and methods

### Materials

The leaves and twigs of *S. brevipes* were collected at the Popa Mountain National Park (GPS location: 20°55′N 95°15′E), Mandalay Prov., Myanmar, in August 2013. Prof. Yong Dong Kim (Hallym University, Chuncheon, Korea) identified the plant. A voucher specimen (number: Cho S.H. et al. MM408) was deposited in the herbariums of Hallym University (Chuncheon, Korea) and the National Institute of Biological Resources (Incheon, Korea). RAW264.7 cells and HEK293T cells were purchased from American Type Culture Collection (ATCC) (Rockville, MD). RPMI1640, DMEM, foetal bovine serum (FBS), phosphate-buffered saline (PBS) and penicillin–streptomycin solution were purchased from Hyclone (Logan, UT). Lipopolysaccharide, 3-(4-5-dimethylthiazol-2-yl)-2-5-diphenyltetrazolium bromide (MTT), DMSO, quercetin, luteolin, kaempferol, TRIzol, sodium dodecyl sulphate (SDS) and ranitidine were obtained from Sigma-Aldrich (St. Louis, MO). The cDNA synthesis kit was purchased from Thermo Fisher Scientific (Waltham, MA). Forward and reverse primers for iNOS, COX-2 and TNF-α were synthesized by Bioneer (Seoul, Korea). The kit for the luciferase assay was purchased from Promega (Madison, WI). Luciferase plasmids harbouring NF-κB or AP-1 binding promoter sites were used as reported previously (Yang et al. 2014). Polyvinylidene fluoride (PVDF) membrane was purchased from Merck Millipore (Billerica, MA). Antibodies against c-Jun, c-Fos, p50, p65, lamin A/C, β-actin, IRAK1, IRAK-4 and phospho- and total forms of Src, Syk, p85, IκBα, ERK, JNK, p38, TAK1, MEK1/2, MKK4/7 and MKK3/6 were purchased from Cell Signaling Technology (Beverly, MA) and Santa Cruz Biotechnology (Santa Cruz, CA).

### Preparation of the Sb-EE

The leaves and twigs of *S. brevipes* were dried at room temperature and then cut and pulverized. The pulverized plant tissues (125 g) were extracted with 95% ethanol (1500 mL) in an ultrasonic bath, and the extract was evaporated to dryness under reduced pressure to produce a 95% ethanol extract of *S. brevipes* (Sb-EE) (3.7 g). The Sb-EE powder was dissolved in DMSO at a concentration of 200 mg/mL to make a stock solution. The experiment was performed using DMSO (vehicle) control at the same dilution as a negative control.

### Cell culture

To prepare cell culture media, 1% penicillin–streptomycin was added to RPMI 1640 and DMEM supplemented with 10% FBS and 5% FBS, respectively. RAW264.7 and HEK293T cells were cultured using RPMI1640 and DMEM media in a humidified incubator maintained at 5% CO_2_ and 37 °C. Trypsin was used for the subculture of HEK293T cells, and a cell scraper was used to detach RAW264.7 cells from the plate.

### NO assay

RAW264.7 cells were pre-treated with Sb-EE (0, 25, 50, 100, 150 and 200 µg/mL) for 30 min, then stimulated with LPS (1 µg/mL) for 24 h. The supernatant (100 μL) was mixed with 100 μL of Griess reagent, as reported previously (Hossen et al. 2017; Kim et al. 2018). Griess reagent contains 0.2% naphthylethylenediamine dihydrochloride (NEDD) and 2% sulphanilamide in 5% phosphoric acid. The absorbance of this mixture was measured at 540 nm and the concentration of nitric oxide (NO) was calculated by comparison with a standard curve.

### MTT assay

RAW264.7 cells and HEK293 cells were treated with Sb-EE at the indicated concentrations for 24 h. Then, 10 μL of MTT solution was added and incubated for 3 h and the reaction was stopped using stopping solution (15% SDS), as reported previously (Hong et al. 2018). The samples were then incubated overnight. The absorbance of MTT formazan was measured at a wavelength of 540 nm.

### Animals

We purchased C57BL/6 mice for the peritonitis model and ICR mice for the gastritis model Biolink (Chungbuk, South Korea); 6–8 mice per cage were housed in a 12 h light/dark cycle. The care of animals was based on guidelines issued by the National Institute of Health for the Care and Use of Laboratory Animals (NIH Publication 80-23, revised in 1996). The study was conducted according to the guidelines established by the Institutional Animal Care and Use Committee (IACUC) at Sungkyunkwan University. The IACUC number was SKKUIACUC2018-10-16-1.

### High-performance liquid chromatography (HPLC) analysis

To characterize the phytochemical characteristic of Sb-EE, HPLC analysis was performed as described previously (Almela et al. 2006). Quercetin, luteolin and kaempferol were utilized as standard compounds. Solvent A (0.1% H_3_PO_4_ in H_2_O) and solvent B (acetonitrile) were used as elution solvents. For analysis, a system equipped with a KNAUER (Wellchrom) HPLC-pump K-1001, a Wellchrom high-speed scanning spectrophotometer K-2600, and a four-channel deaerator K-500 and a Phenomenex Gemini C18 ODS (5 μm) column was used.

### Semi-quantitative RT-PCR

RAW264.7 cells were pre-treated with Sb-EE for 30 min and then stimulated by LPS (1 µg/mL) for 6 h. Total RNA was isolated using TRIzol reagent according to the manufacturer’s instructions. Semi-quantitative PCR was performed as reported previously (Baek et al. 2016; Lee et al. 2019). The sequences of primers used in this study are listed in Table 1.

**Table 1.** Sequences of PCR primers used in this study.

Targets	Direction	Sequences (5′ to 3′)
iNOS	Forward	GGAGCCTTTAGACCTCAACAGA
	Reverse	TGAACGAGGAGGGTGGTG
COX-2	Forward	CACTACATCCTGACCCACTT
	Reverse	ATGCTCCTGCTTGAGTATGT
TNF-α	Forward	GCCTCTTCTCATTCCTGCTTG
	Reverse	CTGATGAGAGGGAGGCCATT
GAPDH	Forward	CAATGAATACGGCTACAGCAAC
	Reverse	AGGGAGATGCTCAGTGTTGG

### Preparing whole or nuclear lysates

To obtain total cell lysate, harvested cells were first washed with cold PBS containing 1 mM sodium orthovanadate. The washed cells were then lysed in ice-cold modified RIPA buffer (50 mM Tris–HCl, pH 7.4, 1% Nonidet P-40, 0.25% sodium deoxycholate, 150 mM NaCl, 1 mM Na_3_VO_4_ and 1 mM NaF) including protease inhibitors (2 mM PMSF, 100 μg/mL leupeptin, 10 μg/mL pepstatin, 1 μg/mL aprotinin and 2 mM EDTA) for 30 min with rotation at 4 °C. At this time, a sonicator (Thermo Fisher Scientific, Waltham, MA) was used to improve cell lysis efficiency as reported previously (Choi et al. 2019). Lysates were refined by centrifugation at 16,000×*g* for 10 min at 4 °C and stored at −20 °C until use. To prepare nuclear lysate, a three-step procedure was performed. In the first step, harvested cells were washed and lysed with 500 mL lysis buffer (50 mM KCl, 0.5% Nonidet P-40, 25 mM HEPES, 1 mM phenylmethylsulfonyl fluoride, 10 μg/mL leupeptin, 20 μg/mL aprotinin and 100 μM 1,4-dithiothreitol) on ice for 4 min. Second, lysates were centrifuged at 16,000×*g* for 1 min. The pellet obtained from lysates was washed with washing buffer (lysis buffer without Nonidet P-40). Finally, the pellet containing nuclei was lysed with an extraction buffer (lysis buffer with 500 mM KCl and 10% glycerol). The nuclei/extraction buffer mixture was frozen at −80 °C, and then centrifuged at 16,000×*g* for 5 min. The supernatant was collected as a nuclear extract.

### Western blotting

Proteins in total cell lysates or nuclear fractions were separated with 7–15% SDS-polyacrylamide gel electrophoresis (PAGE) (Choi et al. 2019) and then transferred onto a PVDF membrane. To reduce non-specific antibody reactions, the PVDF membrane was first blocked with BSA. After washing twice with Tris-buffered saline including Tween 20 (TBST), the membrane was incubated overnight with primary antibody in BSA. After three washing steps with TBST, the membrane was probed with a secondary antibody conjugated with horseradish peroxidase in BSA for 1 h. Immunoreactive bands were detected using an enhanced chemiluminescence kit (Pierce ECL Western blotting substrate, Thermo Scientific, Waltham, MA). Two different blots were obtained from two independent Western blotting analyses. Band intensity was measured and quantified using ImageJ (Bethesda, MD).

### Luciferase assay

HEK293T cells were transfected with plasmids harbouring a luciferase gene under an NF-κB promoter (NF-κB-Luc) or AP-1 promoter (AP-1-Luc). To activate the luciferase genes, MyD88 or TRIF genes were co-transfected. A beta-galactosidase (β-gal) reporter gene was also co-transfected as a control for normalization of transfection efficiency. Transfections were performed using the polyethylenimine (PEI) method as reported previously (Hossen et al. 2016; Han et al. 2018). The transfected cells were stabilized for 24 h and then treated with Sb-EE for another 24 h. Luciferase activity was measured with a luciferase assay system as described previously (Woo et al. 2005).

### *In vitro* kinase assay

A kinase profiler service from Millipore (Billerica, MA) was used to assess the direct inhibitory effect of Sb-EE against Src, Syk and IRAK1. An Sb-EE stock solution was prepared to 50× final assay concentration in 100% DMSO. The reaction was initiated by incubating with a Mg/ATP mixture. After 40 min at room temperature, the reaction was stopped by adding 0.5% phosphoric acid. Then, 10 μL of the reaction solution was spotted onto a P30 filtermat and washed four times for 4 min in 0.425% phosphoric acid, followed by one wash in methanol. After this, the sample was dried and by scintillation counts.

### LPS-induced peritonitis *in vivo* model

Acute peritonitis mice were generated using C57BL/6 mice (10 mice/group) as previously reported (Yi et al. 2016). Sb-EE (200 mg/kg) suspended in 0.5% sodium carboxymethylcellulose (CMC) was orally administered once per day for five days. Acute peritonitis was induced with an intraperitoneal injection of 1.0 mL (10 mg/kg) LPS on day 4 after Sb-EE administration. Peritoneal fluid was harvested by intraperitoneal lavage using sterile PBS on day 5. In peritoneal macrophages obtained from peritonitis mice, NO production and phosphorylation pattern of target proteins were analysed using an NO assay and immunoblotting, respectively.

### HCl/EtOH-gastritis *in vivo* model

To generate an acute gastritis mice model, ICR mice (four mice/group) were used as previously reported (Yang et al. 2015). Sb-EE (200 mg/kg) or ranitidine (40 mg/kg) was orally injected into ICR mice twice a day for two days. After 1 h of final administration, 60% ethanol in 150 mM HCl was orally injected to induce gastritis. After 1 h, mice were anaesthetized with isoflurane and sacrificed. Subsequently, redness of gastric mucosal lesions was observed.

### Statistical analysis

For the MTT and NO assays, two independent experiments were performed and each experimental group had 10 parallel wells to ensure the reliability of the results. In PCR, each experimental group was performed in triplicate using lysates obtained from three independent experiments. For Western blot analysis, two independent experiments were performed, and band intensity was measured and quantified using ImageJ (Bethesda, MD). Peritonitis and gastritis *in vivo* experiments were performed with 10 or 4 mice per group, respectively. In this study, all data are presented as means ± standard deviation (SD) obtained from each experiment. Analysis of variance (ANOVA) with Scheffe’s *post hoc* test or the Kruskal–Wallis/Mann–Whitney tests was used to compare data and to assess the significance of group differences. A *p* value less than 0.05 indicates statistical significance.

## Results

### Sb-EE inhibits NO production in LPS-stimulated RAW264.7 cells and contains flavonoids such as quercetin, luteolin and kaempferol

**Figure 1.**
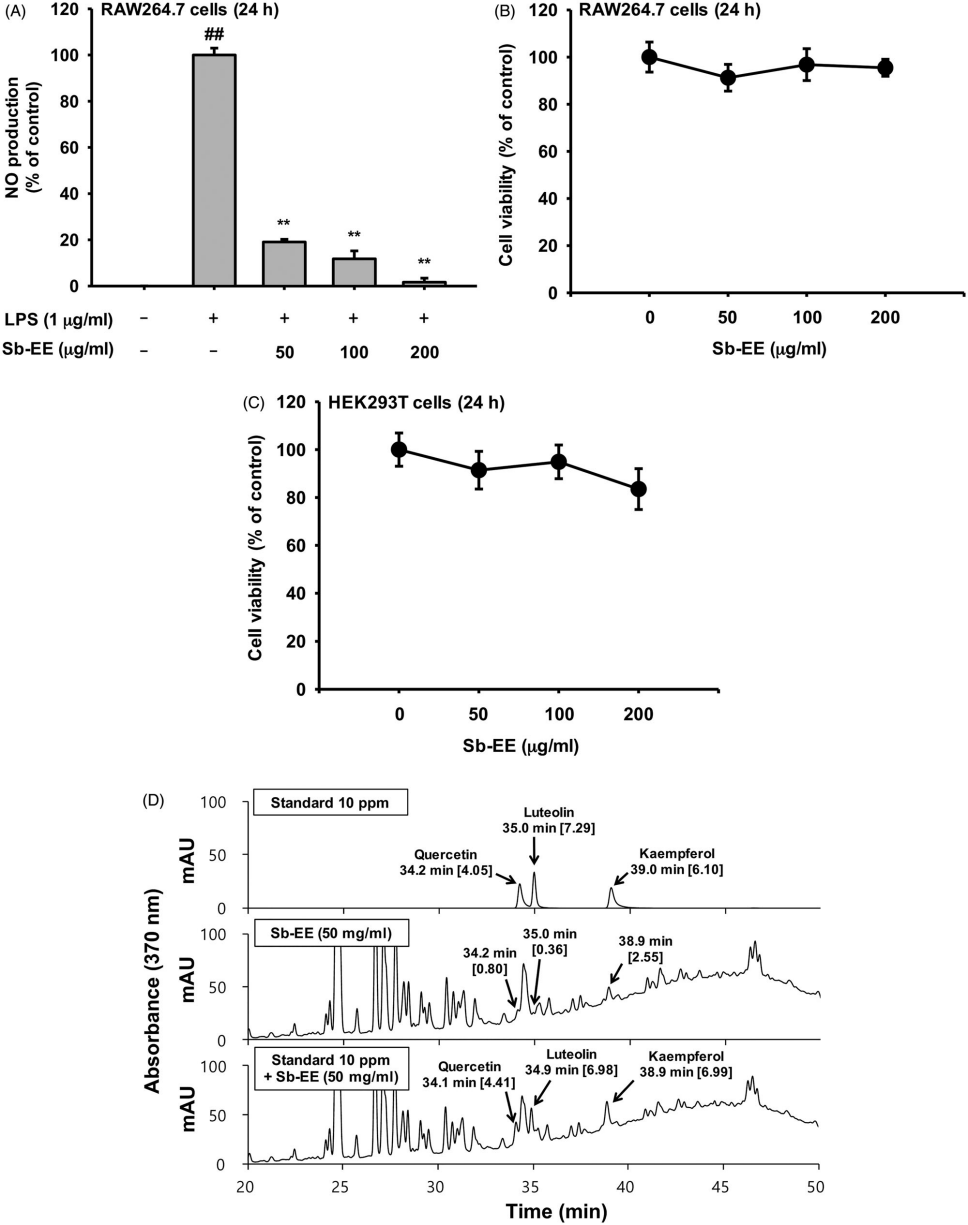
Suppressive activity of Sb-EE against NO production in RAW264.7 cells. (A) To examine the effect of Sb-EE on NO release, Sb-EE pre-treated-RAW264.7 cells were stimulated with LPS (1 µg/mL) for 24 h, and NO levels in supernatant were assessed by NO assay. (B, C) To test the cytotoxicity of Sb-EE, RAW264.7 cells (B) and HEK 293T cells (C) were dose-dependently treated with Sb-EE for 24 h, and then MTT assay was performed. (D) Active phytochemical elements in Sb-EE were assessed by HPLC. The profiles of Sb-EE were compared with standard profiles containing quercetin, luteolin and kaempferol. All data are presented as the mean ± SD of experiments. ^##^*p* < 0.01 compared to the normal group, and **p < 0.01 compared to the LPS-alone treatment group. Rt (min) (area).

To evaluate the anti-inflammatory activity of Sb-EE, we assessed the effect of Sb-EE on NO production in LPS-stimulated RAW264.7 cells. In the Griess assay, Sb-EE (25, 50, 100, 150 and 200 µg/mL) significantly suppressed NO in a dose-dependent manner (IC_50_=34 µg/mL) (Figure 1(A)). On the other hand, MTT assay revealed that Sb-EE did not affect the viability of RAW264.7 and HEK 293 cells (Figure 1(B,C)), which indicates that suppressive effect of Sb-EE on NO production is not due to cytotoxicity. Then, HPLC analysis was performed to identify the phytochemical composition of Sb-EE. Quercetin, luteolin and kaempferol, which have strong antioxidant properties, were used as standard compounds (Crozier et al. 2000; Romanova et al. 2001; Zhang et al. 2011). Peak of each standard compound was observed at certain retention time (quercetin = 34.2, luteolin = 35 and kaempferol = 39), and Sb-EE sample also has peaks at 34.2, 35 and 38.9 min (Figure 1(D)). These results suggest that Sb-EE contained quercetin, luteolin and kaempferol, and that anti-inflammatory activity of Sb-EE may have been derived from these phenolic components.

### Sb-EE suppresses expression of inflammatory genes and the activity of transcriptional factors NF-κB and AP-1

As NO production is mainly regulated by iNOS in macrophages, we evaluated the effect of Sb-EE on *iNOS* gene expression using semi-quantitative PCR (Lirk et al. 2002). To further investigate the anti-inflammatory activity of Sb-EE, we examined the expression patterns of inflammatory genes such as *COX-2* and *TNF-α* in Sb-EE-pre-treated RAW264.7 cells. Sb-EE significantly diminished the mRNA expression of *iNOS* and *COX-2* at all concentrations (50, 100 and 200 µg/mL) in LPS-stimulated RAW264.7 cells (Figure 2(A)). TNF-α expression was also inhibited in the group pre-treated with 200 µg/mL Sb-EE (Figure 2(A)). Then, to understand the molecular mechanism underlying anti-inflammatory activity of Sb-EE, molecular biological assays were conducted using Sb-EE of 200 µg/mL, the most effective but non-cytotoxic concentration. As these genes are regulated at the transcriptional level, we studied whether Sb-EE regulates NF-κB and AP-1, which are major transcriptional factors in inflammatory gene expression (Liu et al. 2018). A luciferase assay was performed using HEK 293 cells susceptible to plasmid transfection. To mimic LPS stimulation in RAW264.7 cells, HEK 293 cells were transfected with MyD88 or TRIF, which are adaptor proteins for TLR4 signalling (Akira and Takeda 2004). We found that Sb-EE blocked only MyD88-dependent NF-κB and AP-1 luc activity, not TRIF-dependent signalling (Figure 2(B,C)). The inhibitory activity of Sb-EE against NF-κB and AP-1 was further confirmed by immunoblotting with nuclear lysates. Consistent with our luciferase assay results, nuclear translocation of NF-κB subunits (p65 and p50) was significantly attenuated by Sb-EE at all indicated times after LPS stimulation (Figure 2(D)). Sb-EE also suppressed nuclear translocation of the AP-1 subunits, c-Fos and c-Jun, 30 min after LPS stimulation (Figure 2(D)). These results indicate Sb-EE exerts its anti-inflammatory properties by inhibiting NF-κB and AP-1 signalling.

Figure 2.Effect of Sb-EE on inflammatory gene expression and transcriptional factors. (A) To verify the effect of Sb-EE on inflammatory biomarker expression, RAW264.7 cells were pre-treated with Sb-EE, then stimulated with LPS (1 µg/mL) for 6 h. mRNA expression of *iNOS*, *COX-2* and *TNF-α* was analysed by semi-quantitative RT-PCR. (B, C) To validate the effect of Sb-EE on the transcriptional activity of NF-κB and AP-1, a luciferase assay was performed. HEK 293 cells were transfected with NF-κB-Luc (B) or AP-1-Luc (C) genes, and Flag-MyD88 or CFP-TRIF were additionally overexpressed to activate transcription of NF-κB-Luc and AP-1-Luc genes. Then, HEK 293 cells were treated with Sb-EE (0–200 μg/mL) for 24 h. Luciferase activity was measured using a luminometer. (D) To analyse nuclear translocation of transcription factor subunits, Sb-EE (200 μg/mL) pre-treated RAW264.7 cells were stimulated with LPS for the indicated times. Then, the levels of AP-1 (c-Fos and c-Jun) and NF-κB subunits (p65 and p50) in nuclear lysate were determined by immunoblotting. Lamin A/C was utilized as a loading control. All data are presented as the mean ± SD of experiments. ^##^*p* < 0.01 compared to the normal group, and *p < 0.05 and **p < 0.01 compared to the control group (LPS-alone group in (A, D), and MyD88/TRIF-alone group in (B, C)).

**Figure:**
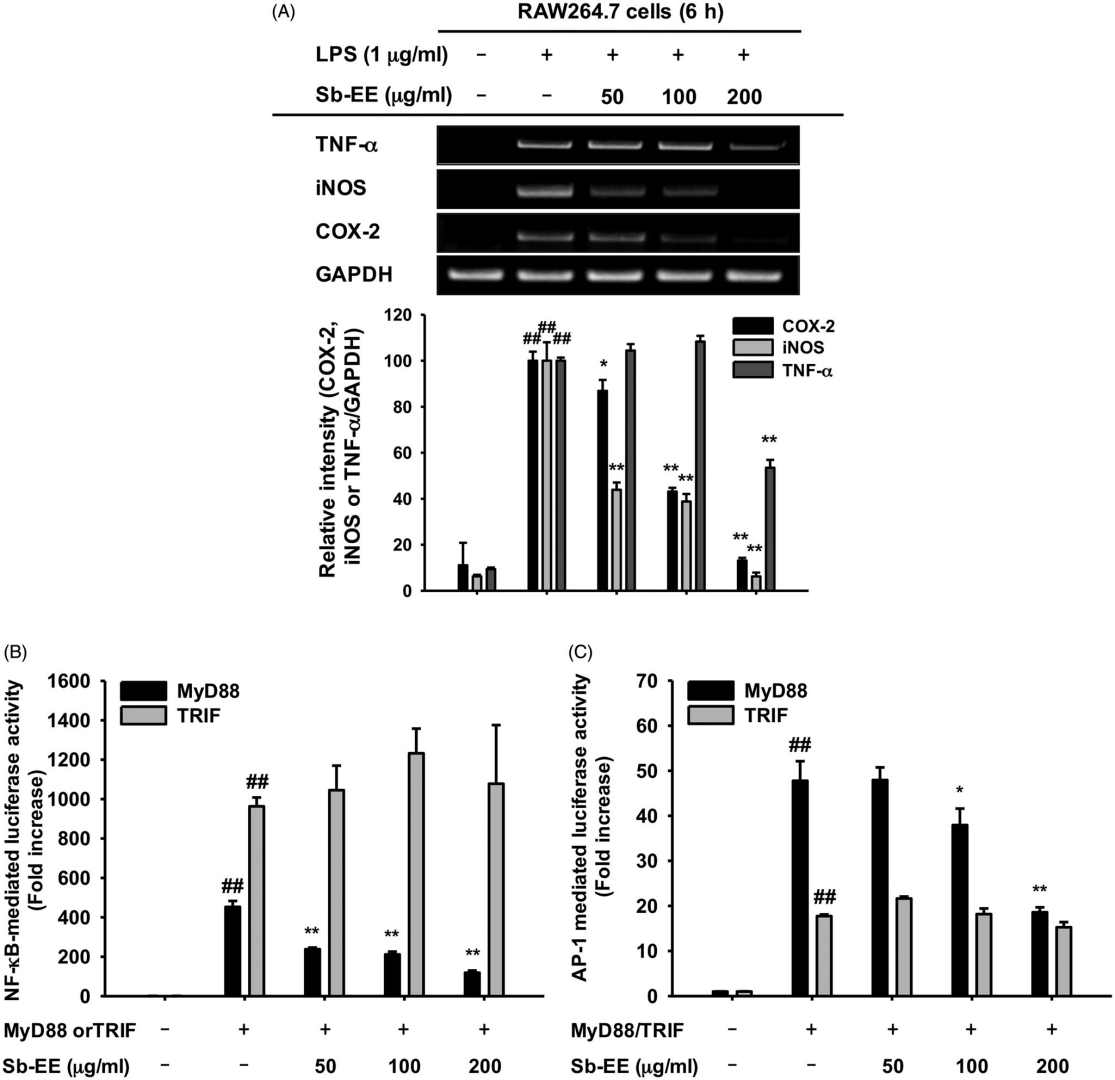

**Figure:**
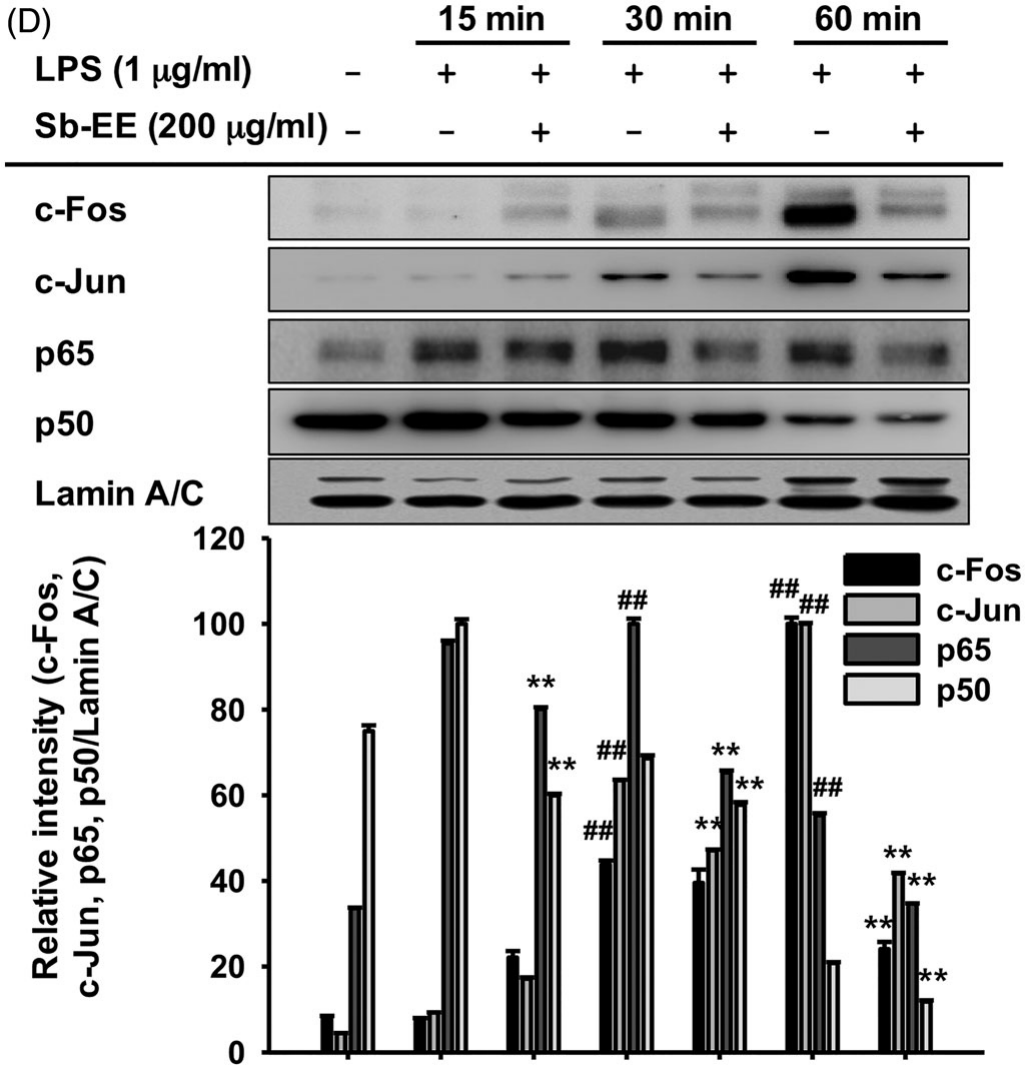

### Sb-EE diminished NF-κB signalling by targeting Src and Syk kinases

**Figure 3.**
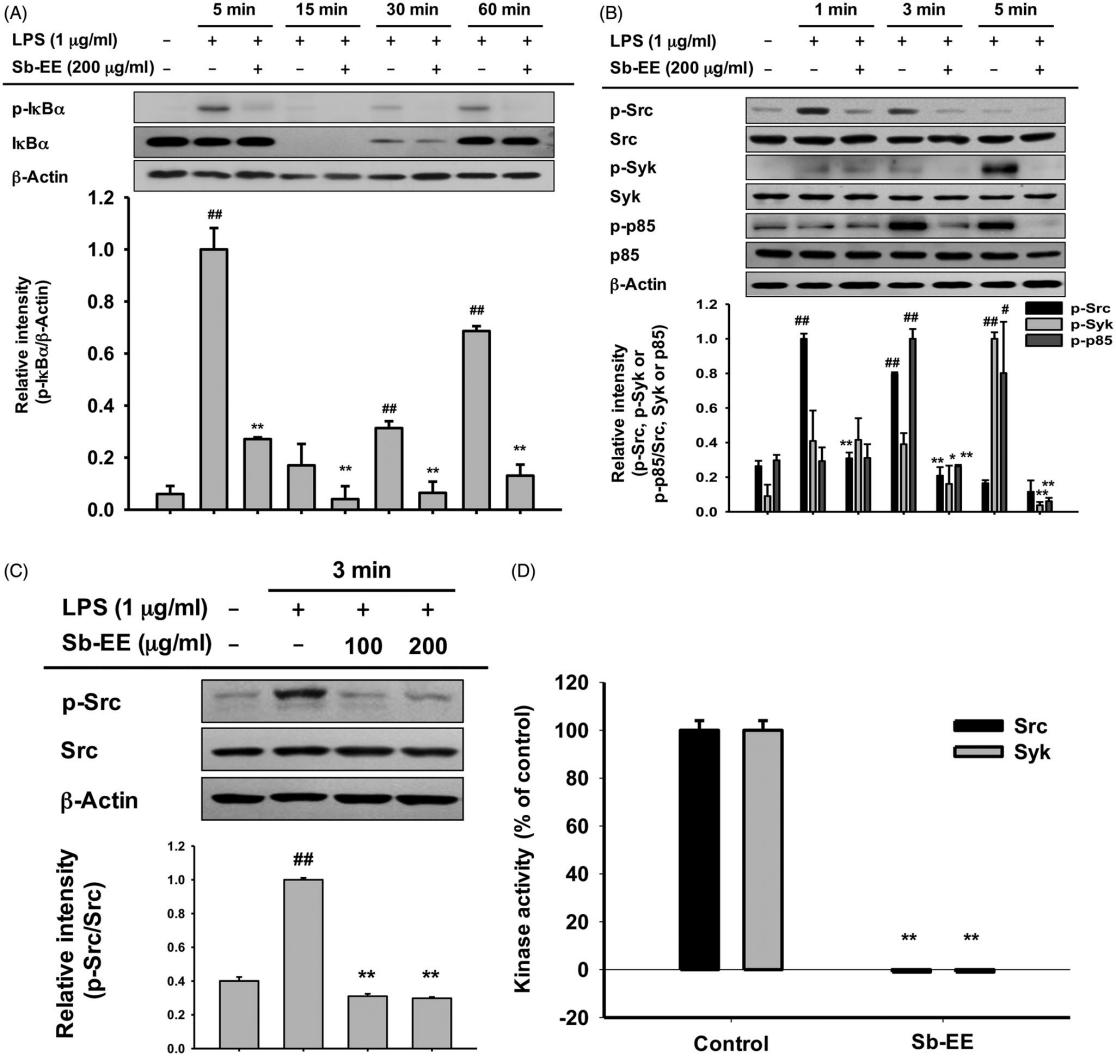
Inhibitory effect of Sb-EE on NF-κB signalling enzymes. (A, B) RAW264.7 cells were treated with LPS (1 µg/mL) for the indicated time in the presence or absence of Sb-EE (200 μg/mL). The levels of phospho- and total-IκBα (A), Src, Syk, p85 and β-actin (B) were determined by immunoblotting using whole lysates. (C) RAW264.7 cells were pre-treated with Sb-EE in a dose-dependent manner, and were thereafter triggered with LPS for 3 min. Then, p-Src, Src and β-actin levels were detected by immunoblotting. (D) To examine the direct effects of Sb-EE on Src and Syk activity, an *in vitro* kinase assay was performed with purified Src and Syk. ^##^*p* < 0.01 compared to the normal group, and *p < 0.05 and **p < 0.01 compared to the control group (LPS alone group in (A–C)).

To understand the molecular mechanism by which Sb-EE regulates the inflammatory response, we examined the alteration in NF-κB signalling enzymes including IκBα, p85, Syk and Src. The phosphorylation levels of each signal molecule were evaluated by immunoblotting in LPS-stimulated RAW264.7 cells after Sb-EE pre-treatment. Sb-EE suppressed p-IκBα levels at all LPS-stimulated time points (5, 15, 30 and 60 min) (Figure 3(A)). Phosphorylation patterns of upstream molecules of IκBα such as p85, Syk and Src were checked at the earlier time than 5 min. As shown in Figure 3(B), p-Syk and p-p85 were inhibited by Sb-EE at 3 min and 5 min after LPS activation. Phosphorylation of Src kinase was also diminished by Sb-EE after 1 min and 3 min of LPS stimulation (Figure 3(B)). Total Src, Syk and p85 were not affected by Sb-EE (Figure 3(B)). Since Src and Syk are the most upstream enzymes in TLR4-mediated NF-κB signalling, we hypothesized that Sb-EE would directly regulate Src and Syk (Lowell 2011) and performed an *in vitro* kinase assay for Src and Syk kinase to test this. As we expected, Sb-EE reduced Src and Syk kinase activity down to basal levels (Figure 3(C,D)). These results imply that Sb-EE directly inhibits Src and Syk kinases to suppress NF-κB signalling.

### Sb-EE attenuates AP-1 signalling by targeting IRAK1

Since Sb-EE suppressed AP-1 luc activity and nuclear translocation of the AP-1 subunit, we explored the effects of Sb-EE on AP-1 signalling regulators, such as ERK, JNK and p38 (Figure 2(C,D)). To identify the target of Sb-EE among the signalling molecules passing through the AP-1 pathway, we stimulated Sb-EE-pre-treated RAW264.7 cells with LPS for various times. Immunoblotting analysis revealed that phosphorylation of ERK is blocked by Sb-EE after 30 min of LPS simulation (Figure 4(A)). Sb-EE also inhibited phosphorylation of JNK and p38 five min after LPS treatment without changing the total levels of each enzyme (Figure 4(A)). Phosphorylated MEK1/2, an upstream regulator of ERK, was inhibited by Sb-EE at 15 and 30 min after LPS stimulation. In the case of MKK4/7, a JNK regulator, and MKK3/6, a p38 activator, phosphorylation was suppressed 5 min after the LPS trigger (Figure 4(B), left and right panel). As TAK1 is an indispensable kinase that activates MKK4/7 and MKK3/6 in the TLR-AP-1 signal pathway (Kishimoto et al. 2000), we evaluated whether Sb-EE controls TAK1. Sb-EE blocked p-TAK1 levels at all indicated time points (5, 15, 30 and 60 min) in LPS-triggered RAW264.7 cells (Figure 4(B), left and right panel). We further assessed modulation of IRAK1/4, the most upstream regulators of the TLR4-dependent AP-1 pathway (Gan and Li 2006). Interestingly, LPS-induced degradation of IRAK1 was restored by Sb-EE at 3 min, which suggests that IRAK1 might be a target of Sb-EE (Figure 4(C)). Our hypothesis was verified by *in vitro* kinase assay. As we expected, Sb-EE decreased the kinase activity of IRAK1 by 68% (Figure 4(D)). These results indicate that Sb-EE directly inhibits IRAK1, leading to an anti-inflammatory response.

Figure 4.Inhibitory effect of Sb-EE on AP-1 signalling enzymes. (A–C) RAW264.7 cells were treated with LPS (1 µg/mL) for the indicated time in the presence or absence of Sb-EE (200 μg/mL). Then, an immunoblotting assay was performed with antibodies for phospho- and total-forms of target proteins. The antibodies against p-ERK, ERK, p-JNK, JNK, p-p85 and p85 antibodies were used to detect the MAPKs (A). The antibodies against p-TAK1, TAK-1, p-MEK1/2, MEK1/2, p-MKK4/7, MKK4/7, p-MKK3/6 and MKK3/6 were used to detect the upstream enzymes of MAPKs (B, left and right panel). IRAK-1 and IRAK-4 antibodies were used to detect the initially activated enzymes in the AP-1 signalling pathway (C). (D) To examine the direct effects of Sb-EE on IRAK1 activity, an *in vitro* kinase assay was performed with purified IRAK1. ^##^*p* < 0.01 compared to the normal group, *p < 0.05 and **p < 0.01 compared to the control group (LPS-alone group in (A), (B, left and right panel) and (C)).

**Figure:**
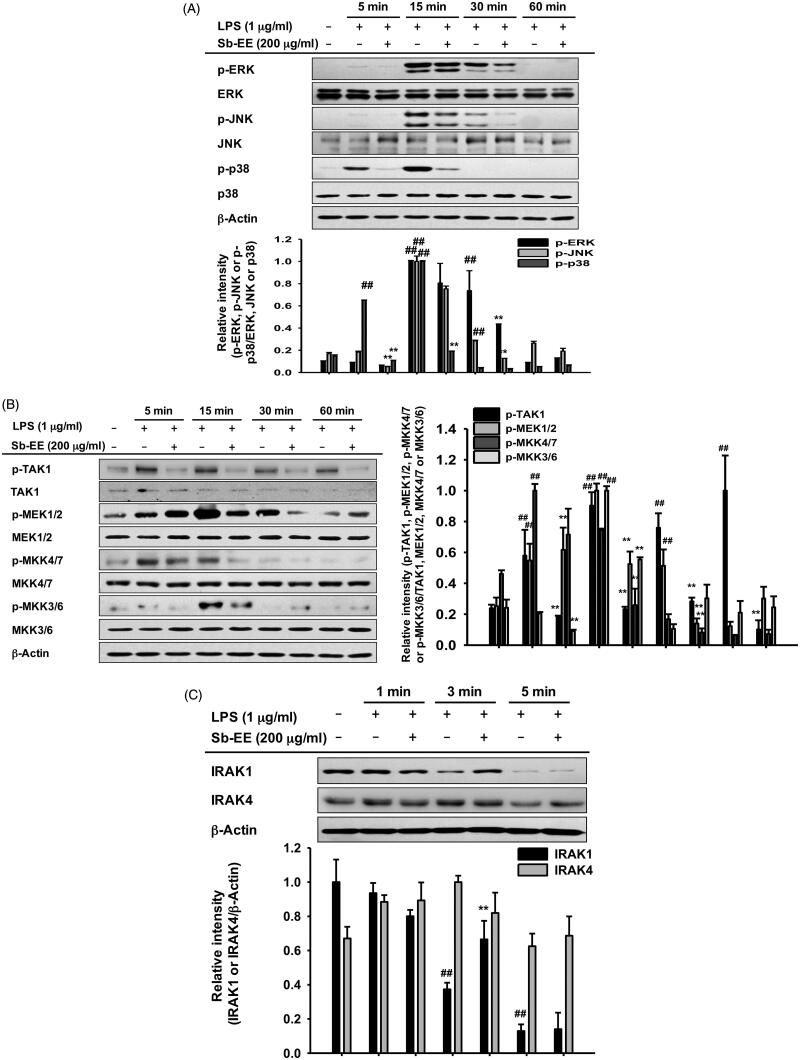

**Figure:**
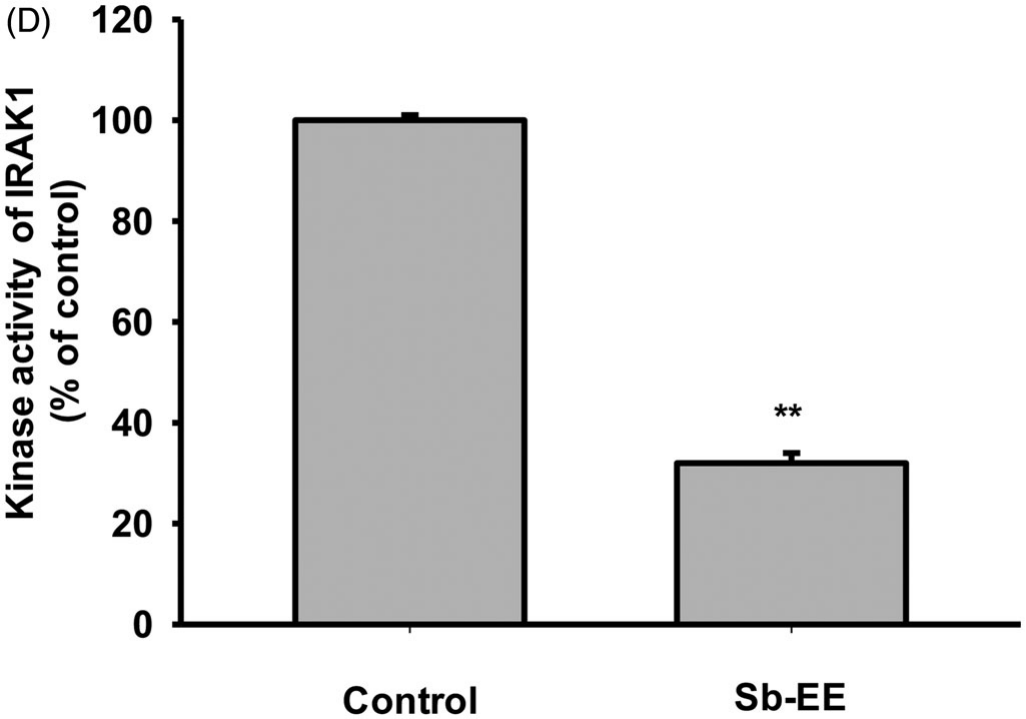

### Sb-EE alleviates LPS-induced peritonitis and HCl/EtOH-stimulated gastritis in mice

**Figure 5.**
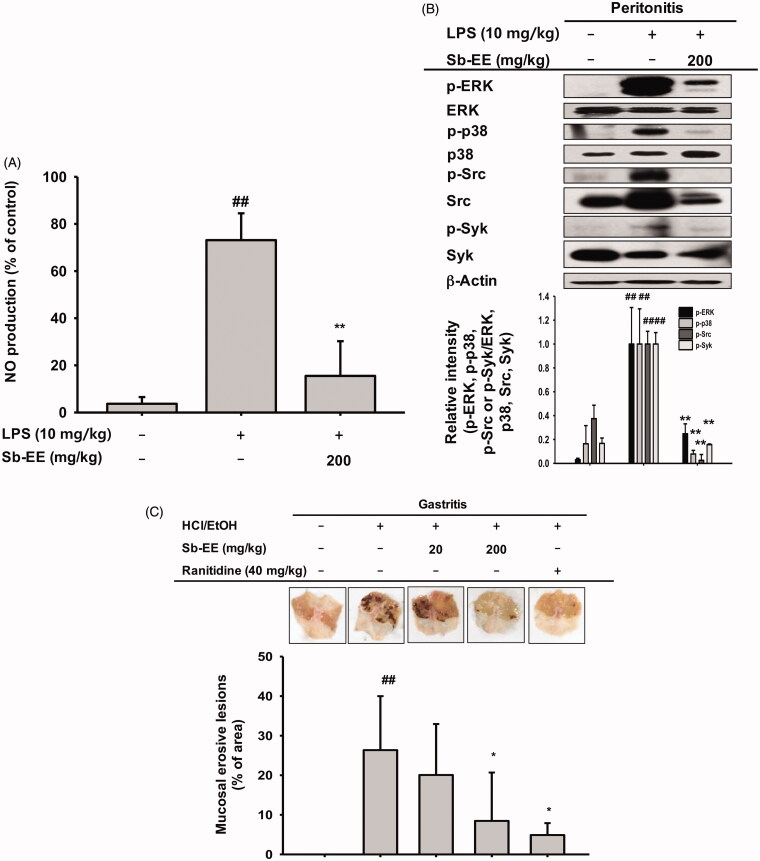
Anti-inflammatory ability of Sb-EE in LPS-induced peritonitis and HCl/EtOH-triggered gastritis mice. (A, B) Sb-EE (200 mg/kg) was orally injected into C57BL/6 mice daily for five days. Peritonitis was induced by peritoneal injection of LPS (10 µg/kg) for one day. NO assay was performed using peritoneal macrophages (A). Immunoblotting assay was performed with whole lysates obtained from peritoneal exudates. Phospho- or total-forms of ERK, p38, Src, Syk and β-actin were detected using specific antibodies (B). (C) Sb-EE (200 mg/kg) or ranitidine (40 mg/kg) was orally administered in ICR mice twice a day for two days, and then HCl/EtOH was injected for 1 h to induce gastritis. Stomach inflammatory lesions were photographed with a digital camera and then quantified using ImageJ. ^##^*p* < 0.01 compared to the normal group, *p < 0.05 and **p < 0.01 compared to the control group (LPS-alone group in (A, B), and HCl/EtOH alone group in (C)).

We further evaluated the anti-inflammatory activity of Sb-EE in *in vivo* peritonitis and gastritis mouse models. Peritonitis was induced by intraperitoneal injection of 10 mg/kg LPS after oral administration of Sb-EE for five days. To assess the inhibitory effect of Sb-EE against peritonitis, we performed an NO assay and immunoblotting analysis with macrophages obtained from the peritoneums of peritonitis-induced mice. The NO assay showed that Sb-EE (200 mg/kg) suppresses NO production in peritonitis mice (Figure 5(A)). In addition, immunoblotting with lysates of peritoneal macrophages revealed that Sb-EE inhibited phosphorylation of ERK, p38, Src and Syk (Figure 5(B)), which is consistent with our *in vitro* results. Next, we induced gastritis in mice by oral administration of HCl/EtOH for 1 h after orally injecting Sb-EE or ranitidine, a positive control, three times at 12 h intervals. We then observed haemorrhagic mucosal damage to determine the degree of gastric mucosal inflammation. Bleeding and redness of the gastric mucosa was reduced in both the ranitidine and Sb-EE-treated groups (Figure 5(C)). These results indicate that Sb-EE is effective in the treatment of inflammatory diseases including peritonitis and gastritis.

## Discussion

The ethanolic extract of *S. androgynous* has been reported to exert anti-inflammatory activity against carrageenan induced rat paw oedema at dose of 400 mg/kg/body weight (Selvi and Anusha 2012). The anti-inflammatory property of this herb was also proved *in vitro* experiment using RAW 264.7 cells (Lee et al. 2011). In the case of *S. rostratus*, water extract of the root shows anti-inflammatory effect on dimethylbenzene-induced auricle oedema mice (Zhen et al. 2013). In this study, we evaluated the pharmaceutical effects of the ethanolic extract of *S. brevipes in vitro* and *in vivo*. As shown in Figure 1(A,B), Sb-EE significantly reduced NO release without cytotoxicity. As NO is a key inflammatory mediator and various anti-inflammatory drugs including NSAIDs target NO synthesis and secretion (Stratman et al. 1997; Di Girolamo et al. 2003), Sb-EE was also expected to have potent therapeutic efficacy in inflammatory diseases. PCR results showing the inhibitory effects of Sb-EE on inflammatory gene expression of *iNOS*, *COX-2* and *TNF-α* also supported our hypothesis (Figure 2(A)). Therefore, we decided to validate the therapeutic effect of Sb-EE on inflammatory diseases using *in vivo* models such as LPS-induced peritonitis mice and HCl/EtOH-triggered gastritis mice. Consistent with RAW264.7 cell results, oral administration of Sb-EE (200 mg/kg) strongly inhibited NO levels in peritoneal macrophages from peritonitis mice (Figure 5(A)). Sb-EE also alleviated gastritis symptoms, which suggests that it can be used as treatment for inflammatory diseases.

Flavonoids are natural substances with a phenolic structure, and widely distributed in plants (Galeotti et al. 2008; Higdon et al. 2017). They exhibit the biological activities such as anticancer, anti-inflammatory and antiviral, which are based on their antioxidant properties (Wei et al. 2011; Zhen et al. 2013; Madhu et al. 2014; Zhang et al. 2017; Kuttinath and Rammohan 2019). Flavonoids are classified into five subtypes according to their structure. Of them, flavones and flavonols with C-2,3-double bonds have been reported to show strong inhibition of NO production (Kim et al. 2004). Since anti-inflammatory activity of flavonoids is primarily due to their scavenging effect on NO (Nijveldt et al. 2001), it was investigated whether Sb-EE contained luteolin, kaempferol and quercetin which are representative flavonones and flavonols. HPLC analysis revealed that Sb-EE contains these standard flavonoids (Figure 1(D)). In addition, Sb-EE inhibited the gene expression of *iNOS* (Figure 2(A)), similar to the results of previous studies that the mechanism of NO inhibition of flavonones and flavonols is dependent to iNOS down-regulation, not to iNOS activity (Kim et al. 2004). This suggests that luteolin, quercetin and kaempferol are the active ingredients of Sb-EE. Furthermore, as several high peaks were observed at retention time of 20–30 min (Figure 1(D)), it is expected that a large number of phytochemicals other than luteolin, kaempferol and quercetin might be contained in Sb-EE. Plants belonging to the genus *Sauropus* have a high content of apigenin which is a powerful NO inhibitor and a natural flavonoid (Bunawan et al. 2015). Therefore, Sb-EE is also expected to contain apigenin, and eventually these flavonoids are expected to synergistically reduce the NO production and to exhibit anti-inflammatory action.

To understand the intracellular mechanism of Sb-EE, we examined alterations in the TLR4 signalling pathway during Sb-EE treatment. Since Figure 2(A) shows a defect in the mRNA expression of inflammatory genes, we confirmed these changes in transcriptional factors. Sb-EE blocked both NF-κB and AP-1 activity (Figure 2(B–D)), and also suppressed phosphorylation of upstream enzymes responsible for activating NF-κB and AP-1 signalling such as IκBα, p85, ERK, JNK, p38, TAK1, MEK1/2, MKK4/7 and MKK3/6 (Figures 3(A,B) and 4(A,B), left and right panel). In TLR4 signalling, external stimuli activate NF-κB and AP-1 through recruitment of the adapter molecules MyD88 and TRIF (Akira and Takeda 2004). Interestingly, Sb-EE affects only MyD88-activated NF-κB and AP-1 luciferase activities, not TRIF-triggered signalling (Figure 2(B,C)), which suggests that the MyD88-dependent pathway is a target of Sb-EE. According to immunoblotting and *in vitro* kinase assays, Sb-EE directly inhibited Syk, Src and IRAK1 kinases, which are activated first in response to PAMPs (Figures 3(B,D) and 4(C,D)) (Kawagoe et al. 2008; Lowell 2011). As TLR4 transduces MyD88 signals via IRAK4/1 to activate AP-1 (Li et al. 2002), inhibition of IRAK1 by Sb-EE correlates with luciferase assay results. On the other hand, the role of Syk and Src kinase is more complicated in intracellular TLR signalling. In the classic TLR4 pathway, Src and Syk tend to be activated first and function together in response to LPS engagement (Lowell 2011). Then, they can activate MyD88 and TRIF signals through different pathways. For example, Src kinase can activate a MyD88 dependent pathway via TRAF6 (Liu et al. 2012). On the other hand, there is also report demonstrating that Src is involved in TRIF-mediated IFN-β production (Li et al. 2017). Syk also promotes internalization of TLR4 by phosphorylation of ITAM motif-containing proteins such as DAP12 and FcεRIγ, thereby activating TRIF-mediated interferon production (Zanoni et al. 2011). As the role of Syk in TRIF signalling is limited to regulating interferon-regulatory factor (IRF), not NF-κB and AP-1, Sb-EE seems to show selectivity for the MyD88-pathway in terms of NF-κB and AP-1 regulation.

## Conclusions

**Figure 6.**
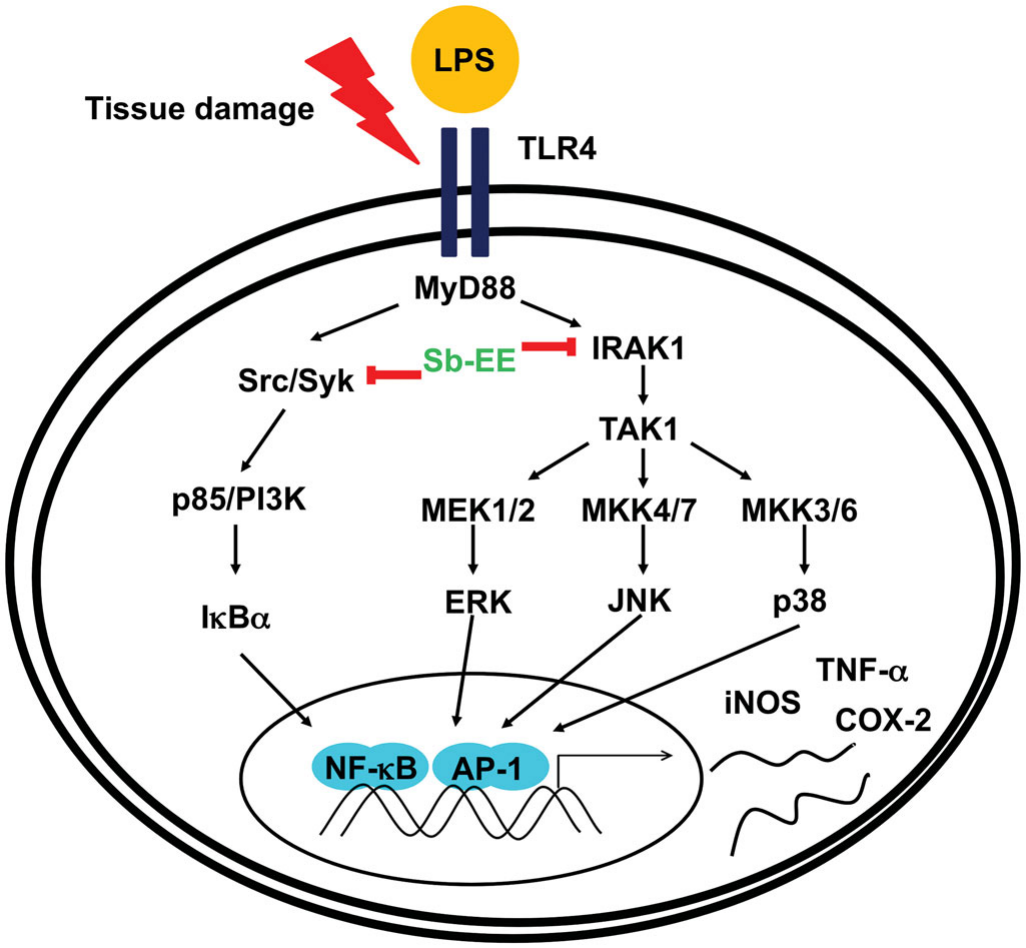
Schematic diagram of the anti-inflammatory properties of Sb-EE through inhibition of AP-1 and NF-κB signalling.

Sb-EE suppressed NO production in RAW264.7 cells and attenuated inflammatory symptoms in LPS-induced peritonitis and HCl/EtOH-triggered gastritis mice, which suggest Sb-EE has anti-inflammatory activity. Sb-EE inhibited phosphorylation of LPS-triggered signal molecules, such as p85, IκBα, TAK1, MEK1/2, MKK4/7, MKK3/6, ERK, JNK and p38. In particular, Sb-EE acts as a direct inhibitor of Src, Syk and IRAK1, thereby inhibiting MyD88-dependent NF-*κ*B and AP-1. As a result, Sb-EE blocked mRNA expression of *iNOS*, *COX-2* and *TNF-α*, which play critical role in inflammation (Figure 6). The paradigm of drug development is changing from single-target to multi-target drugs with the increase in chronic and intractable diseases. Therefore, drug development derived from natural products capable of interacting with various proteins in a biological system is accelerating (Koeberle and Werz 2014). In this respect, it is expected that Sb-EE derived from edible herbs can be used to develop a potent anti-inflammatory agent.
